# Mineral Oil Aspiration Related Juvenile Idiopathic Arthritis

**DOI:** 10.1155/2015/403109

**Published:** 2015-06-10

**Authors:** Andrew D. Nelson, Philip R. Fischer, Ann M. Reed, Mark E. Wylam

**Affiliations:** ^1^Mayo Clinic College of Medicine, Rochester, MN 55905, USA; ^2^Department of Pediatric and Adolescent Medicine, Mayo Clinic College of Medicine, Rochester, MN 55905, USA; ^3^Department of Internal Medicine, Mayo Clinic College of Medicine, Rochester, MN 55905, USA

## Abstract

We describe the development of rheumatoid factor-positive migratory polyarthritis in a 5-year-old male who had been administered bidaily oral mineral oil as a laxative since birth. Minor respiratory symptoms, radiographic and bronchoscopic findings were consistent with chronic lipoid pneumonia. We speculate that immune sensitization to mineral oil promoted the clinical syndrome of juvenile idiopathic arthritis.

## 1. Introduction

Juvenile idiopathic arthritis (JIA) is an autoimmune disorder characterized by chronic inflammation and destruction of cartilage and bone. JIA is caused by both genetic and environmental triggers. There are 17 genes which are linked to the development of the disease [1]. However, genetics alone is not solely responsible for the development of the childhood disease. Environmental factors including maternal smoking [2] and infections such as parvovirus B19 [3] in combination with genes can trigger juvenile arthritis in children. Mineral oil exposure is a well-established model of inducing rheumatoid arthritis in mice [4]. Epidemiologic evidence suggests a strong correlation between mineral oil exposure and incidence of rheumatoid arthritis [5]. Herein we report a case of chronic exogenous mineral oil aspiration and development of refractory rheumatoid arthritis.

## 2. Case Report

A 5-year-old Caucasian boy presented with worsening, episodic leg, and ankle pains since two years of age. Painful episodes lasted several days and were typically intermittent, about every four to six weeks. Increasing frequency and new symptoms in feet and hands prompted referral to our facility. Past medical history was notable for difficulty to gain weight, complex partial seizure disorder, and long-term constipation. The constipation was managed with 5 mL oral mineral oil administered bidaily by syringe since postnewborn age. At age of 5 years, he weighed 19.7 kg (49th percentile) and was 113.2 cm tall (69th percentile). Examination was normal, including lung exam, except for swelling of the dorsum of wrists with decreased dorsiflexion, extension, and pain with motion. Hemoglobin, white blood count, and morphology were all identified within normal ranges. He had elevated rheumatoid factor (RF) at >802 IU/mL, anti-cyclic citrullinated peptide antibody 121 U (strongly positive > 60), positive anti-histone antibody 1.3 U (nl < 1.0 U), erythrocyte sedimentation rate at 27 mm/h, and C-reactive protein (CRP) < 3 mg/L. There were no known cardiac, pulmonary, or renal abnormalities, but outside chest radiography “opacifications” during an evaluation for “pneumonia” were noted.

**Figure 1:**
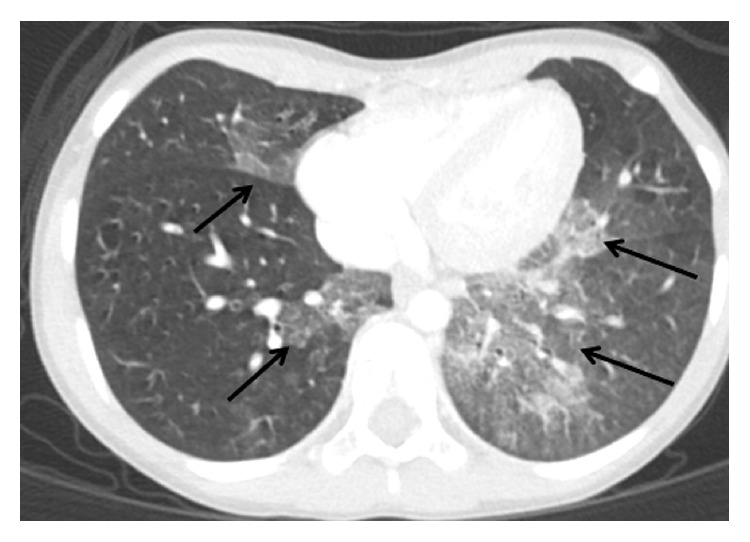
High resolution CT image of lower lobes. Arrows show diffuse hazy mixed alveolar interstitial infiltrates involving much of the left lower lobe and medial portions of the lingula, right middle lobe, and medial right lower lobe.

Magnetic resonance imaging of the right wrist showed diffuse mottled edema and hazy enhancement of carpal bones. There was moderate thickening and enhancement of the synovium and soft tissue surrounding the carpus. Soft tissue wrist biopsy of the extensor sheath showed fibrovascular and synovial tissue with moderate lymphoplasmacytic inflammation. A routine chest radiogram showed a left lower lobe infiltrate. Subsequently, a chest computed tomography scan (Figure 1) revealed a mixed alveolar interstitial infiltrate in left and right lower lobe, lingula, and right middle lobe with a crazy-paving pattern. Fiber optic bronchoscopy demonstrated a normal larynx and tracheobronchial tree. An oil red O cytochemical stain was performed on the bronchoalveolar lavage specimen, which was strongly positive for exogenous lipoid aspiration. Lipid-laden macrophage index was 84%.

The diagnoses were consistent with chronic lipoid pneumonia, a silent complication of chronic occult mineral oil aspiration and inflammatory arthritis consistent with RF positive JIA. Discontinuance of mineral oil and initiation of hydroxychloroquine (100 mg/day) and methotrexate (12.5 mg/week) lead to a moderate improvement in frequency and severity of symptoms. At age of 7 years he has not achieved clinical remission and he continues to have episodic flairs of joint pain leading to school absence and restricted activity.

## 3. Discussion

RF positive JIA is a chronic autoimmune disorder eliciting inflammation and destruction of joints [6, 7]. In childhood the incidence of JIA varies worldwide noting the heterogeneous nature of a disease that is recognized primarily by clinical examination rather than diagnostic testing. It is estimated that in North America and Europe roughly 4 to 16 out of 10,000 children are affected [8].

It is generally accepted that the development of JIA is a consequence of the environmental triggers in a genetically susceptible individual [9]. Gender specific factors such as estrogen may be immunomodulatory. Clearly, HLA and non-HLA susceptibility genes may enhance disease acquisition, severity, and response to treatment. Single gene microsatellites and nucleotide polymorphisms, such as variations in PTPN22, STAT4, and TRAF1-C5, may be independent risk factors [6, 10, 11]. Environmental factors that contribute to adult rheumatoid arthritis (RA) include cigarette smoking and certain infectious agents. Occupational exposure to silica, electrical components, and wood dust may have an increased risk of RA. Our patient had no known family history of RA, exposure to tobacco, or known recent infections prior to the onset or clinical JIA.

In 1956, Pearson demonstrated that complete Freund's adjuvant caused arthritis in animal models [12]. Originally this was felt to be due to the mycobacterium as a consequence of immunity towards mycobacterium spreading to the joints. Later, it was determined that the pathologic T-cell response would also occur due to incomplete Freund's adjuvant, that is, mineral oil alone. Early this millennium, Holmdahl et al. [4] showed that exposure to mineral oil results in an increased number of T-cells in murine joints. In laboratory models of RA mineral oil is injected intradermally with an emulsifier which can cause polyarthritis in 100% of male and female DA rats, but not in Lewis rats [11]. Micelle transport of mineral oil to local lymph nodes initiates a generalized cytokine response as well as IL-23 triggering synovial fibroblasts activation [4, 13]. Intranodal T-cells multiply rapidly and express the T-cell receptor (*αβ*TCR) which binds to antigens located on major histocompatibility complex (MHC) molecules found in joints [4].

Exogenous lipoid pneumonia occurs due to the inhalation or aspiration of fatty substances, chiefly mineral oil. Clinical symptoms include cough, dyspnea, chest pains, hemoptysis, or periodic fevers. These symptoms are likely due to direct an inflammatory response or coexisting infection. Common substances aspirated to cause lipoid pneumonia include petroleum jelly, that is, Vaseline, mineral oils, navel drops, and animal fats. Lip balm, lip-gloss, olive oil, and sesame oils are also risk factors despite being less common. In adults some cases of lipoid pneumonia [14] have been reported due to nasal instillation of oils or occupational inhalation/aspiration of fatty substances such as Parafilm, transmission fuel, machine oil, and spray paint [15–17].

In the past chronic childhood constipation was often managed with regular administration of mineral oil. However, this routine practice has been abandoned due to the high incidence of lipoid pneumonia [18]. Indeed, in our case lipoid pneumonia was not initially suspected as the pulmonary symptoms were nonspecific. Additionally, many patients show no symptoms and lipoid pneumonia is only diagnosed due to abnormalities found in chest radiographs [18, 19].

Mineral oil entering the tracheobronchial tree does not promote the cough reflex, inhibits mucociliary transport system, and becomes long-term resident within alveoli. As macrophages are incapable of metabolizing the mineral oil this results in lipid granulomatous inflammation and alveolar fibrosis.

In our case the chest CT showed a dependent lung zone “crazy-paving” pattern consistent with lipoid pneumonia. Moreover, the bronchoalveolar lavage showed significant oil red O within alveolar macrophages consistent with chronic mineral oil aspiration. The history of long-term bidaily mineral oil use was undoubtedly the cause of the lipoid pneumonia. Indeed we speculate that the sustained long-term use of mineral oil elicited an autoimmune JIA. We suggest that clinicians continue to strongly discourage mineral oil use as a cathartic and investigate its possible occult use in very young children with JIA.
